# Special Issue “Genomics of Stroke” 2022

**DOI:** 10.3390/genes14020514

**Published:** 2023-02-17

**Authors:** Svetlana A. Limborska, Ivan B. Filippenkov

**Affiliations:** Institute of Molecular Genetics of National Research Center “Kurchatov Institute”, Kurchatov Sq. 2, 123182 Moscow, Russia

Stroke is one of the greatest medical threats to human health and quality of life in modern society. This disease regularly ranks as one of the top causes of the general mortality of the population [1,2,3]. Stroke is an example of a multifactorial disease, which is still characterized by an acute shortage of effective methods of diagnosis, prevention and treatment. The genomic approaches to the problem of stroke are one of the most significant and are aimed at personifying solutions. Considering the parameters of genomic architecture and activity inherent in an individual or population, much more effective recommendations for measures to prevent events and negative outcomes of stroke can be formed.

Stroke genomic studies are being actively carried out in several directions at once. First, the search for genomic markers of stroke is carried out depending on the ethnic group of the patient and the etiology of the disease. Second, the regulation of gene expression under conditions of ischemia (including experimental models) and corrective (therapeutic) influence is being studied. Third, translational studies are being carried out to ensure the application of the genomic approach to practical medicine. Started in 2021, this Special Issue (SI), entitled “Genomics of Stroke”, provides a platform for papers related to all major directions of the genomic study of stroke.

In 2022, this SI collection was continued. Thus, this editorial prefaces the second part of the SI «Genomics of Stroke». This part of the SI includes one review and five research articles.

The results and prospects for stroke genomics were systematized and discussed in a substantial review entitled “Stroke and Etiopathogenesis: What Is Known?” by Ciarambino et al. [4]. In this review, the authors summarized the latest evidence until February 2022 of ischemic stroke genetics that may be of interest to the physician and useful for day-to-day clinical work in terms of both the prevention and treatment of ischemic stroke. The authors elaborate on the topic of the association between genetic alterations and risk factors of both monogenic and polygenic cerebrovascular diseases [4]. A significant part of the review is devoted to epigenetic causes of stroke, including DNA methylation, histone modifications, as well as non-coding RNA functioning [4]. The authors concluded that the knowledge of stroke-risk loci increases the possibility of obtaining new drug targets for antithrombotic therapy, thus highlighting the potential of stroke genetics in the field of drug discovery and laying the foundations for an understanding, in a more concrete way, of the intimate relationship that exists between the genetic characteristics of each individual and stroke [4].

The research articles in the 2022 collection of our SI are focused on genomic and post-genomic studies of stroke, using patients and model animals.

First, a paper entitled “Insight into Glyproline Peptides’ Activity through the Modulation of the Inflammatory and Neurosignaling Genetic Response Following Cerebral Ischemia–Reperfusion”, by Stavchansky et al. [5], refers to pharmacogenomic research. In this study, the authors use three Pro-Gly-Pro (PGP)-containing peptides (Met-Glu-His-Phe-Pro-Gly-Pro, named by Semax; PGP; and Pro-Gly-Pro-Leu (PGPL)) to differentiate their effects in a rat transient middle cerebral artery occlusion (tMCAO) model [5]. It should be noted that biologically active natural and synthetic peptides represent potential drugs for reducing the damage that occurs after ischemia [6,7]. The PGP tripeptide that is produced by the intra- and extracellular catabolism of collagen, elastin, and related proteins is an active factor of resistance to the biodegradation of peptide drugs [8]. In a paper by Stavchansky et al., using real-time reverse transcription PCR, the effect of peptides on the expression of a number of genes in the inflammatory cluster (IC) and a neurotransmitter cluster (NC) was studied 24 h after tMCAO. Furthermore, a gene enrichment analysis was carried out, and a regulatory gene network was constructed. As a result, the comparison of glyproline peptides allowed the authors to determine their general and individual effects on gene expression under cerebral ischemia [5]. The study can be significant for the creation of peptide drugs that are even more effective than those currently available.

The two following articles use large model animals (a monkey and a pig). These studies are extremely interesting from the standpoint of translating the results to the clinical level.

A paper entitled “Expression of Transcription Factor ZBTB20 in the Adult Primate Neurogenic Niche under Physiological Conditions or after Ischemia”, by Stoyanov et al. [9], is related to the study of regeneration processes in the brain, which are significant in ischemia. Namely, the authors studied the expression pattern of transcription factor ZBTB20 in the adult primate anterior subventricular zone (SVZa) and rostral migratory stream (RMS) of a macaque monkey by means of immunofluorescence, as well as through the analysis of images derived from a public database of gene expression [9]. Ischemia is known to be a strong promoter of progenitor proliferation in the monkey SVZa [10]. Stoyanov et al. demonstrated enhanced postischemic ZBTB20 mRNA levels in parallel with an increased percentage of ZBTB20 co-expression with Ki67 and DCX markers. These data suggest that ZBTB20 is a candidate regulator of primate SVZa precursor cell proliferation [9].

Moreover, Fedulova et al. presented their paper entitled “Proteomic Markers in the Muscles and Brain of Pigs Recovered from Hemorrhagic Stroke” [11]. The authors used a model of left-sided intracerebral hematoma in pigs. It should be noted that the use of pigs as a model for studying stroke is due to the similarity of their neurophysiological processes with humans [12]. Fedulova et al. carried out trypsinolysis of tissue proteins and chromato-mass-spectrometric analysis (HPLC-MC) of the obtained peptides [11]. As a result, proteins were identified that are expressed during the recovery period after traumatic injury. Based on proteomic results, a regulatory network was constructed for proteins involved in the regulation of some biological processes in studied tissues under intracerebral hematoma model conditions [11]. The results obtained are significant to establish postgenomic and biochemical relationships, in order to understand the biological mechanisms associated with recovery after hemorrhagic stroke.

Fusco et al. presented their paper entitled “Transcriptome Analysis Reveals Altered Expression of Genes Involved in Hypoxia, Inflammation and Immune Regulation in Pdcd10-Depleted Mouse Endothelial Cells” [13]. The authors carried out genome-wide RNA sequencing (RNA-Seq) and a quantitative polymerase chain reaction (Q-PCR) validation analysis in Pdcd10-silenced and wild-type mouse endothelial cells, in order to better elucidate cerebral cavernous malformations (CCM) molecular pathogenesis [13]. It should be noted that CCMs are common vascular malformations derived from capillaries and small vessels of the central nervous system and are often present with stroke [13,14]. Moreover, alterations of PDCD10 are the rarest genetic cause of family CCM and tend to associate with a more aggressive phenotype with an earlier age of onset [15]. Among differentially expressed genes, Fusco et al. revealed the major cluster fell in signaling related to inflammation and pathogen recognition, including HIF1α and Nos2 signaling and immune regulation [13]. Thus, the study by Fusco et al. allowed novel Pdcd10-controlled molecular pathways to be identified and offered the possibility of providing novel insights into family CCM pathogenesis and therapeutic targets [13].

Finally, we consider a paper entitled “Genotype-Phenotype Correlation and Functional Insights for Two Monoallelic TREX1 Missense Variants Affecting the Catalytic Core” by Amico et al. [16]. The authors reported the clinical–neuroradiological features of two patients with Aicardi–Goutières Syndrome (AGS)-like (Patient A: White female at the age of 2 years and 7 months) and cerebral autosomal dominant arteriopathy with subcortical infarcts and leukoencephalopathy (CADASIL)-like (Patient B: White male at the age of 55 years) phenotypes carrying the heterozygous p.A136V and p.R174G variants of the three-prime repair exonuclease 1 (TREX1) gene, respectively [16]. Previously, it was shown that in mammalian cells, TREX1 exonuclease degrades DNA to prevent aberrant nucleic-acid sensing [17,18]. Moreover, excessive activation of the cGAS-STING pathway in patients affected by TREX1 mutations leads to the abnormal secretion of type-I interferon (IFN) and nucleic-acid driven inflammation [19,20]. Moreover, heterozygous missense or frameshift TREX1 mutations have been shown to be attributed to cerebrovascular diseases. In the present study, Amico et al. showed that while the p.A136V variant was unlikely to be causative for AGS in Patient A, Patient B’s phenotype was potentially related to the p.R174G variant. Concomitantly, Amico et al. clarified that further functional investigations of TREX1 variants found in CADASIL-like patients are warranted to determine any causal link and interrogate the molecular disease mechanism(s) [16].

In conclusion, the diversity and quality of the works presented in this SI indicate the constant advancement of knowledge in the field of stroke genomics.

We hope that the second (2022) part of our SI will be useful and interesting for readers. Further development of the field of stroke genomics will move society closer to improving diagnostic, prognostic, and therapeutic measures to combat stroke and related pathologies.
